# Frutapin, a lectin from *Artocarpus incisa* (breadfruit): cloning, expression and molecular insights

**DOI:** 10.1042/BSR20170969

**Published:** 2017-07-21

**Authors:** Felipe Domingos de Sousa, Bruno Bezerra da Silva, Gilvan Pessoa Furtado, Igor de Sa Carneiro, Marina Duarte Pinto Lobo, Yiwei Guan, Jingxu Guo, Alun R. Coker, Marcos Roberto Lourenzoni, Maria Izabel Florindo Guedes, James S. Owen, David J. Abraham, Ana Cristina de Oliveira Monteiro-Moreira, Renato de Azevedo Moreira

**Affiliations:** 1 Northeast Biotechnology Network (RENORBIO), Centre of Experimental Biology (Nubex), University of Fortaleza (UNIFOR), CEP 60811-905, Fortaleza-Ceará, Brazil; 2 Department of Biochemistry and Molecular Biology, Federal University of Ceará (UFC), Campus do Pici s/n, Bloco 907, CEP 60451-970, Fortaleza-Ceará, Brazil; 3 Laboratório de Biotecnologia e Biologia Molecular, Northeast Biotechnology Network (RENORBIO), State University of Ceará (UECE), CEP 60714-903, Fortaleza-Ceará, Brazil; 4 Fiocruz, Fundação Oswaldo Cruz - Ceará, Drugs and Biopharmaceuticals Development Group: Evolution, *in silico* and *in vitro* of Biomolecules, CEP 60175-047 Fortaleza, CE, Brazil; 5 Division of Medicine, The Wolfson Institute for Biomedical Research, University College London, Gower Street, London WC1E 6BT, U.K.; 6 Division of Medicine, Institute of Liver and Digestive Health, University College London, Royal Free Campus, London NW3 2PF, U.K.; 7 Division of Medicine, Centre for Rheumatology and Connective Tissue Diseases, University College London, Royal Free Campus, London NW3 2PF, U.K.

**Keywords:** biotechnology, computational biochemistry, protein-carbohydrate interactions

## Abstract

*Artocarpus incisa* (breadfruit) seeds contain three different lectins (Frutalin, Frutapin (FTP) and Frutackin) with distinct carbohydrate specificities. The most abundant lectin is Frutalin, an α-D-galactose-specific carbohydrate-binding glycoprotein with antitumour properties and potential for tumour biomarker discovery as already reported. FTP is the second most abundant, but proved difficult to purify with very low yields and contamination with Frutalin frustrating its characterization. Here, we report for the first time high-level production and isolation of biologically active recombinant FTP in *Escherichia coli* BL21, optimizing conditions with the best set yielding >40 mg/l culture of soluble active FTP. The minimal concentration for agglutination of red blood cells was 62.5 µg/ml of FTP, a process effectively inhibited by mannose. Apo-FTP, FTP–mannose and FTP–glucose crystals were obtained, and they diffracted X-rays to a resolution of 1.58 (P2_1_2_1_2_1_), 1.70 (P3_1_21) and 1.60 (P3_1_21) Å respectively. The best solution showed four monomers per asymmetric unit. Molecular dynamics (MD) simulation suggested that FTP displays higher affinity for mannose than glucose. Cell studies revealed that FTP was non-cytotoxic to cultured mouse fibroblast 3T3 cells below 0.5 mg/ml and was also capable of stimulating cell migration at 50 µg/ml. In conclusion, our optimized expression system allowed high amounts of correctly folded soluble FTP to be isolated. This recombinant bioactive lectin will now be tested in future studies for therapeutic potential; for example in wound healing and tissue regeneration.

## Introduction

The genus *Artocarpus* (Moraceae) comprises approximately 50 species of evergreen and deciduous trees and economically is an important source of fruit and timber. It includes mainly jackfruit (*Artocarpus integrifolia*) and breadfruit (*Artocarpus altilis*, also known as *Artocarpus incisa*) trees, which are restricted to evergreen forests in the humid tropical zone [1,2]. The genus is also widely used in folk medicines, prompting scientific interest in secondary metabolites possessing useful biological activities.

*Artocarpus integrifolia* seeds contain three lectins with distinct affinities for carbohydrates: Jacalin (D-galactose binding lectin), Artocarpin (D-mannose binding lectin; previously called ArtinM or KM+) [3] and Jackin (chitin-binding lectin) [4]. Lectins are the proteins with specific recognition and reversible binding to carbohydrates or glycoconjugates. This function allows many different interactions on cell surfaces and often confers lectins with both inflammatory and anti-inflammatory properties, as well as immunostimulatory actions [5].

In *Artocarpus incisa* seeds, we found lectins with characteristics similar to those in jackfruit seeds, the most abundant being Frutalin, a multiple-binding lectin recognizing a range of different ligands, though with higher affinity for α-D-galactose moieties [6,7]. Next in abundance was Frutapin (FTP), which we characterize herein, and finally, Frutackin (a chitin- binding lectin). All three share high sequence similarity to the corresponding lectins in jackfruit seeds. Frutalin has 98% amino acid similarity to Jacalin, while FTP is a homologue of Artocarpin, a lectin with strong affinity for D-mannose that was successfully cloned and expressed in *Escherichia coli* [8]. Although these lectins share high structural homology, biologically they exhibit particular activities. Frutalin is able to specifically recognize prostate cancer tissues when compared with Jacalin [9]. Artocarpin, through interaction with N-glycans of toll-like receptor 2 (TLR2) stimulates macrophages and dendritic cells to produce IL-12 [10,11], and also induces both macrophage proliferation and neutrophil haptotactic migration [12]. In addition, Artocarpin accelerates the process of wound healing and epithelial tissue regeneration [13], confirming its potential for biomedical applications and as a pharmaceutical.

Based on such attractive properties of Artocarpin, we resolved to obtain recombinant FTP, the predicted mannose-binding lectin from *Artocarpus incisa* seeds. Although we had isolated native FTP 15 years ago, further studies proved problematic; the multiple-binding lectin, Frutalin, was a consistent contaminant, yields were extremely low and the FTP N-terminal was blocked [14]. Here, we report heterologous expression and production of soluble biologically active FTP in *E. coli* and also describe the crystal structure of Apo-FTP plus changes on binding α-D-glucose or α-D-mannose.

## Results

### Cloning, expression and purification of FTP

**Figure 1:**
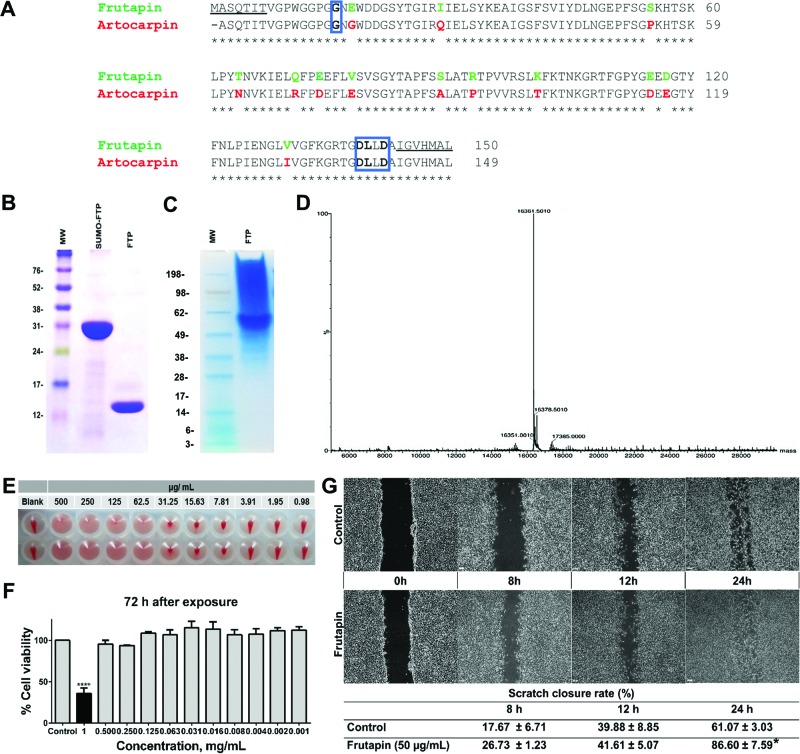
Expression, purification and biological activity of recombinant FTP (**A**) Sequence alignment between FTP and Artocarpin (Q7M1T4, UniProtKB) using Clustal Omega with the N- and C-terminal amino acids used in primer design for *FTP* gene cloning underlined. The expanded carbohydrate binding site for each protein is boxed in blue with carbohydrate interacting residues shown in bold. (**B**) After cleavage of the SUMO-tag, purified recombinant FTP showed a single band by SDS/PAGE. (**C**) Native gel electrophoresis also showed a single band of 58.3 kDa, as measured by an Rf compared with log_10_ (MWt) plot, consistent with an FTP tetramer. (**D**) FTP gave a mass of 16.3 kDa by deconvoluted MS. The recombinant FTP demonstrated biological activity as judged by its (**E**) haemagglutination activity (agglutinated duplicate wells are translucent whereas red blood cells precipitated to form a red dot in the absence of agglutination), (**F**) lack of cytotoxicity towards cultured mouse 3T3 fibroblast cells and (**G**) ability to stimulate 3T3 fibroblast cell proliferation in the scratch wound assay.

Attempts to extract RNA from seeds with three different commercial kits gave poor yields and quality, but isolation of RNA from leaves with Qiagen’s RNeasy Plant Mini Kit allowed cDNA synthesis of FTP. Sequenced clones were 450 bp, and FTP and Artocarpin shared 91% amino acid identity (Figure 1A). Expression of His-tagged FTP from the pET28a *E. coli* vector gave substantial amounts, but accumulated as insoluble protein in all the culture conditions assessed. We switched, therefore, to small ubiquitin-like modifier (SUMO) fusion strategy (Invitrogen) validating the construct, named SUMO-FTP, through restriction analysis and sequencing (results not shown). Optimal expression conditions in *E. coli* were: incubation at 20°C, shaking speed 130 rpm, and induction with 0.3 mM IPTG for 16 h, which yielded over 40 mg/l soluble recombinant protein. Protease removal of the 11-kDa SUMO tag gave highly purified 16.3-kDa FTP with a single band by SDS/PAGE (Figure 1B). Analysis by native gel electrophoresis also gave a single band consistent with a homotetramer structure (Figure 1C), as noted for native Artocarpin [15].

### MS and biological activities

Analysis of the purified FTP by ESI-MS gave a major peak mass consistent with the FTP monomer (16361.5 Da), but also small peaks most likely to be due to some fragmentation into neutral pieces (Figure 1D). In a standard haemagglutination test, the minimum concentration for agglutination (MCA) of FTP was 62.5 µg/ml (Figure 1E), while the inhibiting sugar assay showed 42.8 µg/ml of FTP to be inhibited by 100 mM of glucose and 12.4 µg/ml of FTP by 6.25 mM of mannose (results not shown). Analysis by a carbohydrate/protein ratio gave glucose/FTP and mannose/FTP ratios of 3.8 × 10^4^ and 0.8 × 10^4^ respectively. No cytotoxicity effects of FTP were noted in Balb/c 3T3 murine fibroblast cells at concentrations below 500 µg/ml, even after 72 h (Figure 1F), while in the scratch wound assay, 3T3 cells recovered 86.6% of the denuded area when incubated for 24 h with FTP (50 µg/ml) compared with 61.1% for the control (*P*<0.05) (Figure 1G).

### X-ray diffraction

**Figure 2:**
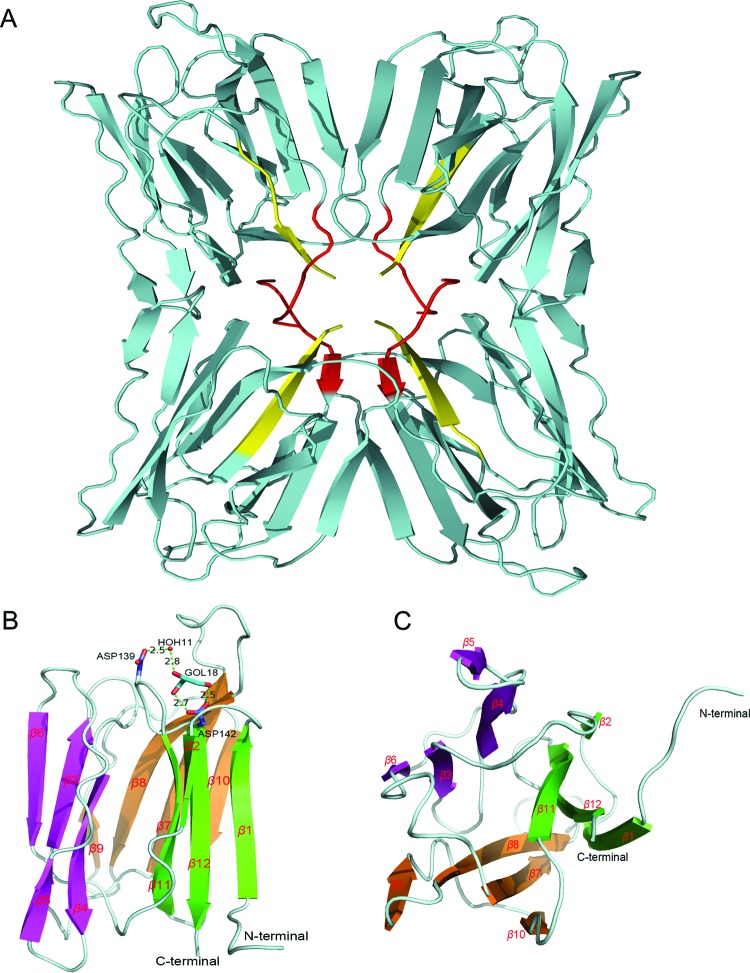
Predicted structural features of FTP (**A**) Secondary structure of tetrameric FTP with the N-terminal in red and the C-terminal in yellow. (**B**) Side view of the structure of one FTP subunit showing the 12 *β*-strands as well as the carbohydrate-binding site with its two key residues, Asp^139^ and Asp^142^. A glycerol molecule attaches to the carbohydrate-binding site forming two direct hydrogen bonds (HBs) with Asp^142^ and one HB with Asp^139^ mediated by a water molecule. (**C**) Bottom view illustrating the *β*-prism I fold composed of three Greek key motifs 1 (green), 2 (purple) and 3 (brown).

Crystals of recombinant Apo-FTP grew in space group P2_1_2_1_2_1_ and diffracted to 1.55 Å at Diamond Light Source (Table 1). Molecular replacement confirmed four monomers in the asymmetric unit and refinement gave a final model with R_factor_: 0.163 and R_free_: 0.200 (Figure 2A). All four monomers are almost identical and their superposition by α carbon (Cα) atoms gave an RMSD of ∼0.15 Å. However, the loops around Leu^90^, which are close to the carbohydrate-binding site, adopt different conformations in different subunits. Analysis using the PDB and PISA server [16–18] suggests that FTP could be a dimer or tetramer in solution with stable interfaces between any of the two relative monomers. A total of 970 water molecules and 20 glycerol molecules were built into the electron density, with one glycerol molecule bound in the carbohydrate-binding site. There are three *cis*-prolines in each monomer all of which fit the electron density well, consistent with homologous structures of FTP (Figure 2B,C) [19,20].

**Table 1:** X-ray parameters for FTP structures

	Apo-FTP	FTP–mannose	FTP–glucose
Beamline	I02 (DLS)	I24 (DLS)	I24 (DLS)
Wavelength (Å)	0.97949	0.96859	0.96859
Space group	*P*2_1_2_1_2_1_	*P*3_1_21	*P*3_1_21
Unit cell parameters			
*a*,*b*,*c* (Å)	67.50, 93.68, 97.74	74.0, 74.0, 185.2	74.0, 74.0, 185.5
*α, β, γ* (°)	90.00, 90.00, 90.00	90.0, 90.0, 120.0	90.0, 90.0, 120.0
Resolution (Å)	38.48 - 1.62	185.24 - 1.70	185.52 - 1.60
	(7.07 - 1.58)	(1.73-1.70)	(1.63-1.60)
*R*_merge_ (%)	11.8 (150.3)	13.3 (90.7)	20.5 (201.8)
*R*_meas_ (%)	14.3 (180.4)	14.5 (99.2)	21.1 (207.3)
*R*_pim_	5.4 (67.9)	5.8 (39.8)	4.8 (47.0)
CC_½_ (%)	99.6 (51.1)	99.2 (61.4)	99.5 (59.4)
Completeness (%)	99.4 (99.7)	100.0 (99.9)	100.0 (100.0)
Average *I*/*σ*(*I*)	8.6 (1.2)	8.8 (2.1)	9.3 (1.9)
Multiplicity	6.9 (6.9)	6.3 (6.3)	19.3 (19.5)
Number of observed reflections	585955 (43074)	413433 (21868)	1516226 (74130)
Number of unique reflections	85385 (6203)	65624 (3461)	78721 (3806)
Wilson plot *B*factor (Å^2^)	19.5	15.76	18.61
*R*-factor (%)	16.3	14.10	14.91
Free *R*-factor (%)	20.0	19.08	19.18
RMSD bond lengths (Å)	0.0204	0.0262	0.0334
RMSD bond angles (°)	1.948	2.477	2.903
Number of reflections in working set	85305	65526	78591
Number of reflections in test set	4087	3342	3913
Mean protein *B* factor (Å^2^)	24.6	21.2	16.4
Solvent content (%)	46.30	43.26	43.45

Values in parentheses are for the outer resolution shell. Abbreviation: DLS, Diamond Light Source.

**Figure 3:**
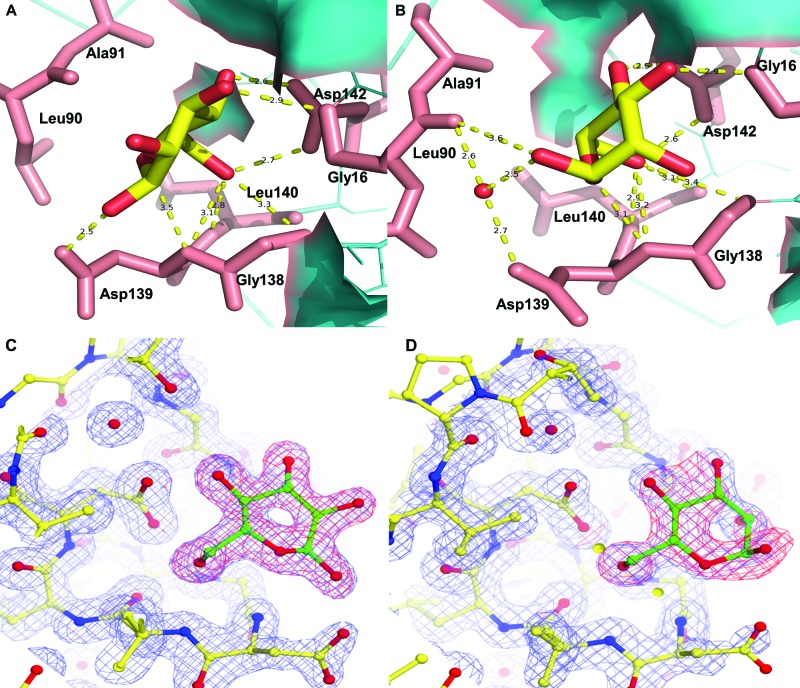
FTP carbohydrate-binding sites with glucose or mannose In the glucose complex (**A**) hydrogen bonding of the C1 hydroxyl group of the glucose to the Asp^139^ carboxylate distorts the sugar ring away from the stable chair conformation, seen for mannose in the FTP–mannose complexes (**B**), towards a more energetically unfavourable boat-like conformation. The 2Fo-Fc maps (blue) and omit maps (red) for the glucose-bound (**C**) and mannose-bound (**D**) structures are also shown.

FTP was also co-crystallized with mannose and glucose; the crystals diffracted to a resolution of 1.70 and 1.60 Å respectively. Although these grew in similar conditions to the Apo-crystals, data processing revealed a different space group: P3_1_2_1_ rather than P2_1_2_1_2_1_. FTP–mannose crystals were twinned by merohedry with a twinning fraction of 22.4%. Both ligand structures showed clear electron density for their respective sugars in the carbohydrate-binding sites of all the four molecules in their asymmetric units, but did not present relevant structural differences when compared with Apo-FTP protein. The binding of both glucose and mannose to FTP is dominated by hydrogen bonding with the sugar hydroxyl groups O3, O4, O5 and O6. In the FTP–glucose complex, an additional HB is formed between the O1 hydroxyl group with the carboxylic side chain of Asp^139^, which appears to induce strain in the sugar ring forcing it out of the more stable chair conformation (Figure 3A–D).

### Molecular dynamics simulations

The molecular dynamics (MD) in aqueous solution present the four monomers in the homotetrameric structure of FTP named Chain A, Chain B, Chain C and Chain D, and the residence time for both glucose and mannose monomers in each are displayed in Table 2. As indicated, they do not remain in the carbohydrate-binding site throughout the whole time of simulation, apart from Chain A for both FTP–glucose and FTP–mannose complexes (Supplementary Video Files S1 and S2 respectively).

**Table 2:** Residence time (ns) of each glucose or mannose monomer in the carbohydrate-binding site formed by four FTP monomers

	Chain A	Chain B	Chain C	Chain D
FTP–glucose	200.0	110.0	2.0	125.0
FTP–mannose	200.0	2.0	75.0	190.0

The Cα backbone co-ordinates of Apo-FTP, FTP–glucose and FTP–mannose were regarded as initial starting structures for calculating the RMSD. The relative stability of FTP structure was related to the RMSD as a function of time. After reaching equilibrium (50 ns), it was noted that the FTP–glucose complex changed from the starting structure at 3.9 ± 0.6 Å, while FTP–mannose was at 2.4 ± 0.2 Å (Table 3). Moreover, RMSDs were recorded for each monomer of FTP. This revealed average RMSD values lower than 2.0 ± 0.1 Å, indicating that the tertiary structure was conserved. RMSDs of Chain A in FTP–glucose and FTP–mannose were lower than 1.5 Å, while the other monomers could be lower than 2.0 ± 0.1 Å, showing slight differences in structural stability between them.

**Table 3:** RMSD of atomic positions calculated for FTP as a homotetramer, for each monomer and its loop

	Tetramer and monomers RMSD (Å)					Loop RMSD (Å)			
	Tetramer	Chain A	Chain B	Chain C	Chain D	L-Chain A	L-Chain B	L-Chain C	L-Chain D
FTP–glucose	3.9 ± 0.6	1.5 ± 0.2	1.9 ± 0.3	2.0 ± 0.1	1.9 ± 0.1	1.4 ± 0.4	3.3 ± 1.1	2.8 ± 0.4	2.9 ± 0.5
FTP–mannose	2.4 ± 0.2	1.7 ± 0.2	1.7 ± 0.2	1.9 ± 0.2	1.4 ± 0.1	1.6 ± 0.4	2.8 ± 0.8	2.8 ± 0.7	2.6 ± 0.6

RMSDs were calculated for FTP as a homotetramer and for each monomer (Chain A, Chain B, Chain C and Chain D). The RMSD of the loop around Leu^90^ and residues 84 and 97 in each of the four monomers (L-Chain A, L-Chain B, L-Chain C and L-Chain D) is also shown. The Cα atoms of amino acids were considered for the overlap and calculation of RMSD.

RMSDs were also calculated for loop regions around Leu^90^ between 84 and 97 residues to the four monomers (L-Chain A, L-Chain B, L-Chain C and L-Chain D). L-Chain A loops from both FTP complexes were the least flexible, remaining steady during the simulation and reaching values lower than 1.6 ± 0.4 Å. L-Chain B, L-Chain C and L-Chain D in both the complexes reached average RMSD values higher than 2.6 Å. The highest mean RMSD, and S.D., was observed for the L-chain B of FTP–glucose (3.3 ± 1.1 Å), reflecting prolonged residence of glucose (up to 75 ns) in the carbohydrate-binding site with an RMSD of 2.0 ± 0.4 Å. After this time, the RMSD increased to 3.8 ± 0.6 Å, implying that the loop changed its position upon glucose exit from the binding site.

**Figure 4:**
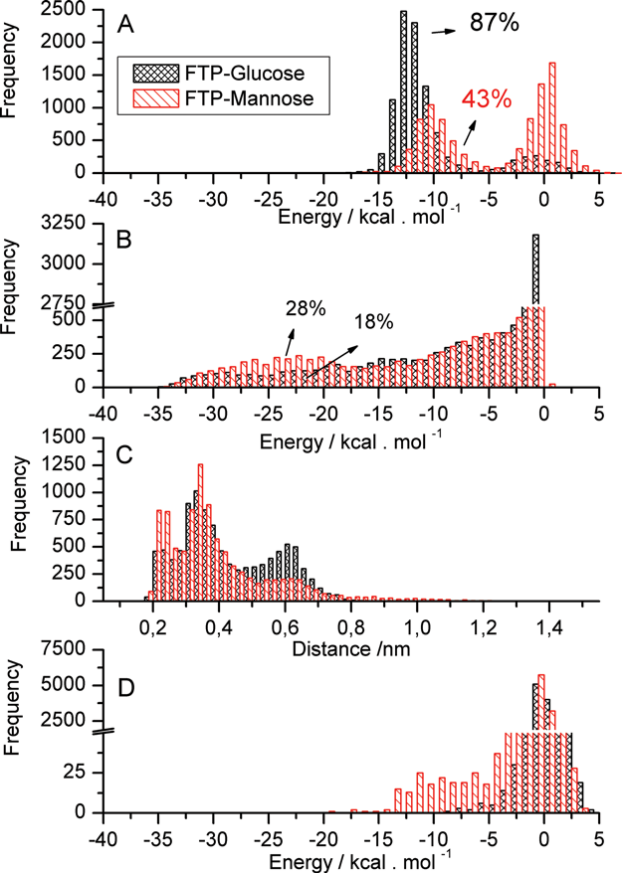
IIP distribution and FTP–glucose (black) and FTP–mannose distances (red) (**A**) IIP between Asp^139^ and sugar monomers, (**B**) IIP between Asp^139^ and Lys^60^, (**C**) Minimal distance among side backbones of Asp^139^ and Lys^60^, (**D**) IIP between Lys^60^ and sugar monomers.

The distribution of the intermolecular interaction potential (IIP) between Asp^139^ to mannose and glucose was obtained during MD simulations (Figure 4). The IIP plot between Asp^139^ and sugar monomers showed two dense regions: (i) with values lower than –5 kcal.mol^−1^ and (ii) from –5 to approximately 5 kcal.mol^−1^. The positive values for IIP indicate that Asp^139^ may interact repulsively with mannose and glucose while in the carbohydrate-binding site. This repulsion is more frequent for mannose than glucose, as observed in the frequencies for energy >0 in IIP (Figure 4A). The integration of values less than the –5 kcal.mol^−1^ limit implies that for 43% of the simulation time, mannose is in appropriate conformation to interact with Asp^139^, while the value for glucose is 87%. Figure 4B shows the IIP distribution between Asp^139^ and Lys^60^ with energies between approximately –35 and 0 kcal.mol^−1^ with a minimum defined at approximately –17.5 kcal.mol^−1^ for FTP–glucose and FTP–mannose. Values recorded below –17.5 kcal.mol^−1^ reveal the formation time percentage of salt bridges between Asp^139^ and Lys^60^ in FTP–glucose (18%) and FTP–mannose (28%). These percentages correspond to distances of 1.8 and 3.2 Å, which allow the formation of hydrogen bonds (HBs) and maintenance of salt bridges between these amino acids residues (Figure 4C). Interaction between Lys^60^ and sugar monomers occurs with low frequency, as indicated by the IIP distribution in Figure 4D. However, it is apparent that IIP is more frequently attractive for FTP–mannose between approximately –17.5 and –5 kcal.mol^−1^ than for FTP–glucose whose energy is greater than approximately 7.5 kcal.mol^−1^.

To understand the dynamical properties of the interfacial HBs, their average number was calculated after 50 ns in the MD simulation trajectory. We observed that Chain A in both FTP–glucose and FTP–mannose complexes gave higher residence time values; both glucose and mannose remained 100% of the MD simulation time (200 ns) in the carbohydrate-binding site. Next, as summarized in Table 4, we made detailed analyses of the interaction and average orientation of glucose and mannose in the carbohydrate-binding site, considering only the sugars in Chain A. This recognition of glucose and mannose residues by FTP is mainly through their backbone group, rather than side chain group interactions. Mannose is slightly more hydrated than glucose (HBs =2.9 compared with 2.6) and its position and conformation in the carbohydrate-binding site allows a considerable number of HBs (3.5) to promote a high protein–sugar interaction compared with glucose (3.0 HBs formed). These HBs are a result of interactions among amino and carboxy groups from Leu^90^, Gly^138^, Asp^139^ and Leu^140^, except Leu^90^ in the case of glucose which occurs via a carbonyl group (C=O). The Asp^139^ residue is on the carbohydrate-binding site surface and plays a key role in anchoring of the sugars. Thus, a glucose–Asp^139^ (carboxylic group, C–O^−^) interaction establishes 0.7 HB with (hydroxyl) H–O1 (C–O^−….^H–O1), while with mannose it is 0.2 HB. Another residue important in maintaining the binding site is Asp^142^, which forms 0.2 HB with both (C–O^−….^H–O4) and (C–O^−….^H–O6) groups in glucose, whereas with the same hydroxyl groups in mannose 0.2 and 0.4 HBs are formed respectively.

**Table 4:** The numbers of HBs in FTP Chain A residues that interact with glucose or mannose

*Groups*	Chain A	
	Glucose	Mannose
*Side chain*		
Asp^139^	(C–O^−….^H–O1) 0.7	(C–O^−….^H–O1) 0.2
Asp^142^	(C–O^−….^H–O4) 0.2	(C–O^−….^H–O4) 0.4
	(C–O^−….^H–O6) 0.2	(C–O^−….^H–O6) 0.2
**Sum side chain**	**1.1**	**0.9**
*Backbone*		
Leu^90^	-	(C=O^….^H–O1) 0.2
Gly^138^	(N–H^….^O6–C) 0.2	(N–H^….^O6–C) 0.3
Asp^139^	(N–H^….^O4–C) 0.7	(N–H^….^O5–C) 0.5
	(N–H^….^O6–C) 0.2	(N–H^….^O6–C) 0.6
Leu^140^	(N–H^….^O6–C) 0.8	(N–H^….^O6–C) 1.0
**Sum backbone**	**1.9**	**2.6**
**Sum groups**	**3.0**	**3.5**

Number of HBs for Chain A residues of FTP that interact with sugars during their residence time within the carbohydrate-binding site. The HBs are represented between the pairs of dipoles formed by the atoms of the protein and the sugar, and involve the carboxy dipole in the aspartic acid residue (C–O^−^), the backbone amino dipole (N–H) and the backbone carbonyl dipole (C=O). The nomenclature of sugar atoms follows that defined by IUPAC, where the sugar dipoles are defined as C–O*n* and H–O*n* with *n* as the atom number of the sugar. Only HBs above 0.2 are shown.

## Discussion

Purification of native FTP from plant extracts is hampered by its low abundance relative to Frutalin, a lectin recognizing different sugars with the same binding site. We opted, therefore, to use heterologous expression in *E. coli* to ensure large-scale, cost-effective isolation as the first step in evaluating FTP for biomedical research, biotechnology applications and medical use. This was not straightforward; initial expression of His-tagged FTP from the pET28a vector produced only insoluble protein, but fortunately, use of the N-terminal SUMO fusion system generated soluble protein which could be readily purified with high yields.

Each lectin molecule possesses two or more carbohydrate-binding sites that can agglutinate cells or react with complex carbohydrates. Such interactions involve displacement of water molecules associated with polar groups of the lectin protein and sugar, and establishment of new HBs; these latter bonds and van der Waals contacts are the dominant forces in binding stability [21,22]. In some cases, cell surface lectins bind to particular glycoproteins, in other cases carbohydrates of cell surface glycoproteins or glycolipids serve as binding sites for bioactive lectin molecules with carbohydrate specificity [5]. In solution, most Jacalin-related lectins are tetramers allowing them to agglutinate erythrocytes even at low concentrations. Alterations in this structure might lead to reduced agglutination ability and carbohydrate recognition, which is recognized for recombinant forms [23], and may explain the relatively high MCA for FTP. Regarding inhibitory sugars in FTP-induced haemagglutination, the lower quantity of mannose needed for inhibition implies that FTP has greater affinity for mannose than glucose. Although many plant lectins bind simple sugars such as glucose, mannose or galactose, they often have much higher affinity for oligosaccharides [24]. Artocarpin has low affinity for simple sugars, interacting preferentially with mannotriose or mannopentose, which suggests the lectin predominantly binds to more complex glycans, perhaps those on cell surfaces of plant pathogens or predators [25].

The FTP structure showed a β-prism I fold (Figure 2B,C), which is found in Jacalin, Artocarpin and other lectins [19] giving a high structural similarity; except for a few loop areas around residues Leu^90^, Glu^36^ and Lys^106^, some of which are flexible and adopt different conformations, even in the same protein. Superposition of the FTP structure by Cα atoms of homologous structures, such as Artocarpin, champedak mannose binding (CMB) and MornigaM lectins, give RMSD values ranging from 0.25 to 0.44 Å [19,20,26]. However, superposition of galactose-binding lectins from *Artocarpus* genus give higher RMSDs, including those for Jacalin (0.77 Å), champedak galactose binding (0.78 Å) and Frutalin (0.87 Å).

Like most-known plant lectins, FTP is a hololectin defined as a multisubunit protein containing several carbohydrate-binding sites to allow cell agglutination. Moreover, these carbohydrate-binding domains are identical or very homologous, and bind either the same or structurally similar sugars. Lectins such as Jacalin and the galactose-binding lectin from champedak fruit bind carbohydrates at one primary and two secondary binding sites [20,25]. In contrast, FTP binds carbohydrates such as mannose and/or glucose in a different way, paralleling CMB [20] and also Artocarpin [20] presumably reflecting the high sequence identities of 90 and 91%, respectively. In Artocarpin, the carbohydrate-binding residues are Gly^15^, Asp^138^, Leu^139^ and Asp^141^ in the binding site formed by a few loops connecting the strands *β*5 and *β6, β*7 and *β*8, *β*11 and *β*12. The equivalent residues in CMB and FTP are the same four, namely Gly^16^, Asp^139^, Leu^140^ and Asp^142^. Several HBs are formed between the mannose molecule and the side chains of these residues in CMB. Interestingly, in our Apo-FTP structure, there is a glycerol molecule (GOL18–a constituent of the crystallization condition) bound at the carbohydrate-binding site, forming two HBs bonds with the carboxylic side chain of Asp^142^ and one HB mediated by a water molecule with the carboxylic side chain of Asp^139^.

Hydrogen bonding is the most dominant interaction in recognition of sugar molecules by the lectin carbohydrate-binding site via formation of HBs with carbonyl and hydroxyl groups of backbone and side chains [27]. The crystal structures of FTP–glucose and FTP–mannose complexes also show this feature: HBs occur between Gly^16^, Leu^90^, Gly^138^, Asp^139^, Leu^140^, Asp^142^ and O3, O4, O5, O6 of the carbohydrates, but they vary slightly both in number and strength from subunit to subunit. In the FTP–mannose structure, the sugar ring is in the energetically favourable boat conformation. However, for FTP–glucose the C1–OH group of the sugar ring is equatorial rather than axial with the sugar ring in the energetically unfavourable chair conformation (Figure 3A,B), which is stabilized by an HB between the C1 hydroxyl group (O1) and the carboxy side chain of Asp^139^. We consider that this difference in sugar ring conformation accounts for FTPs decreased affinity for glucose compared with mannose in the haemagglutination assay.

Through MD simulations in aqueous solution, Lys^60^ found in the loop close to Asp^139^ is forming salt bridges in the FTP–glucose complex (average time of 18%) and also in FTP–mannose (28%), where this salt bridge helps to reduce the interaction between Asp^139^ and mannose, minimizing the repulsion of the mannose hydroxyl groups with oxygen. The Asp^139^–Lys^60^ saline bridge is less frequent in FTP–glucose which enhances the attractive interaction between glucose and Asp^139^ compared with mannose (Figure 4A). By studying crystals of the FTP–mannose complex, a water molecule was found between mannose and Asp^139^ (Figure 3B) consistent with their lower interaction. On the other hand, Lys^60^ interacts more effectively with mannose than glucose, reaching values of interaction potential between –20 and –10 kcal.mol^−1^, while for glucose the potential is > –10 kcal.mol^−1^ (Figure 4D). These values of IIP could suggest that Lys^60^ has a greater influence on extracting mannose from the carbohydrate-binding site than glucose. However, this interaction occurs due to the proximity of Lys^60^ to mannose as a function of the salt bridge with Asp^139^, and more important is that the mannose is anchored in the carbohydrate-binding site forming 3.5 HBs, while the glucose forms 3.0 HBs. These HBs are formed primarily with the amino and carboxy groups from Leu^90^, Gly^138^, Asp^139^ and Leu^140^ (2.6 HBs for mannose and 1.9 for glucose). In case of FTP–mannose, HBs were detected between Leu^90^ (C=O) and mannose (H–O1), an interaction not observed in FTP–glucose. Therefore, mannose is better surrounded in the carbohydrate-binding site and is also stabilized by indirect interaction with Asp^139^ through water molecules, which helps to stabilize the sugar in the binding site. This local structuring is more stable in the case of mannose than glucose indicating higher affinity of FTP for mannose than glucose.

In conclusion, glucose or mannose residues on cell surfaces can act as structural altering agents for FTP, and hence have significance for biomedical applications. Thus, after we had verified low cellular cytotoxicity of FTP, our finding that this lectin stimulates migration of 3T3 fibroblasts in a standard assay implies that future biological investigations are warranted to assess its therapeutic potential, for example in wound healing. Such studies are now underway.

## Materials and methods

### Cloning, expression and purification of recombinant FTP

*Artocarpus incisa* seeds and leaves were collected in Maranguape, Ceará-Brazil for total RNA extraction (RNeasy Plant Mini Kit; Qiagen) and cDNA synthesis (Superscript First Strand Synthesis System; Invitrogen). Primers for FTP cDNA amplification were based on the gene sequence of ArtinM [28] once. Usually these lectins are well conserved in N- and C-terminal regions. The restriction sites are presented (in bold) for BamHI and HindIII (New England Biolabs); forward: 5′-**GGATCC**ATGGCGAGCCAGACGATAACAG, reverse: 3′-**AGCTT**CTAAAGTGCCATGTGAACGCCAATAG. The cloned insert was sequenced (Applied Biosystem 3500 Genetic Analyzer) and the synthetic FTP ORF coding purchased from GeneArt Gene Synthesis (Invitrogen) to obtain the optimized gene sequence for expression in competent *E. coli* cells. Following cloning and chemical synthesis, the *FTP* gene was subcloned into pET28a and the vector transformed into *E. coli* BL21 (DE3). Cells were then inoculated on LB plates supplemented with kanamycin (50 μg/ml), followed by incubation overnight at 37°C.

Positive clones were confirmed using colony PCR. One was used to obtain a 5-ml pre-inoculate of LB medium, which in turn was used to inoculate (1:100) 100 ml LB medium for culturing at 37°C until an OD_600 nm_ of 0.4–0.6 was reached. Aliquots (10 ml) were collected, increasing amounts of IPTG added (0.01, 0.1, 0.3, 0.5 and 1.0 mM) and each culture was incubated at different temperatures (20, 25, 30 or 37°C), under shaking speeds of 200 rpm for 4 or 8 h. Cells were pelleted at 8000×***g*** for 15 min at 4°C, the supernatant discarded and the cells resuspended in lysis buffer (20 mM Tris/HCl, 200 mM NaCl, 200 μg/ml lysozyme and 1 mM PMSF, protease inhibitor). After 15 min, 1% Triton X-100 was added and the homogenates submitted to five cycles of freezing-thawing before centrifugation to separate soluble and insoluble protein fractions. Both parts were quantified (Bradford assay) and analysed by SDS/PAGE. However, as the pET28a vector expressed mainly insoluble FTP, we also cloned the *FTP* gene into the pET SUMO vector (Invitrogen) and essentially repeated the above studies in *E. coli* B21 cells, except that two shaking speeds were used (130 and 200 rpm) and incubation times increased (12, 16 or 21 h).

The SUMO-FTP fusion protein was purified from soluble lysates by immobilized metal affinity chromatography (IMAC) using a HisTrap HP (nickel-sepharose) column equilibrated with wash buffer (50 mM Tris/HCl, 300 mM NaCl, 10% (v/v) glycerol and 40 mM of imidazole, pH 8.0). SUMO-FTP protein was eluted by adding 300 mM of imidazole to the same buffer, dialysed (50 mM Tris/HCl, 300 mM NaCl, 10% (v/v) glycerol) and then incubated with Ubl-specific protease 1 (ULP1) from *Saccharomyces cerevisiae* for 3 h at 37°C with slow stirring. The solution was again passed through the HisTrap HP column to capture the His-SUMO fusion tag and ULP1, while the untagged recombinant FTP was recovered in the flow-through buffer. FTP protein was analysed by SDS/PAGE and ESI-MS using a Synapt HDMS mass spectrometer (Waters, U.K.). Acquired MS data were processed using a maximum-entropy technique (MaxEnt) to obtain a deconvoluted spectrum [29].

### Biological activities of recombinant FTP

#### Haemagglutination and inhibitory carbohydrate assays

FTP-induced haemagglutination, and its inhibition by glucose or mannose, were determined as described previously [30]. After 1 h incubation at 37°C, duplicate wells were assessed to determine the MCA, i.e. the lowest lectin concentration that gave visible agglutination. Inhibition of FTP-induced haemagglutination by carbohydrates was determined using double serial dilutions with lectin and glucose or mannose solutions. Sugar concentrations that visibly inhibited agglutination were identified, using PBS blanks as control.

#### Cell cytotoxicity and migration assays

Cultures of mouse 3T3 embryo fibroblasts were used in standard cell cytoxicity tests and also in scratch wound assays to evaluate the ability of FTP to stimulate cell migration. 3T3 mouse embryo fibroblast cells were cultured in high-glucose Dulbecco’s modified Eagle’s medium (Gibco, U.S.A.) supplemented with 10% FBS (Gibco, U.S.A.) and 1% penicillin–streptomycin in a humidified incubator with 5% CO_2_ at 37°C. Cells were seeded at a density of 5 × 10^3^ per well in 96-well plates. The next day, the attached cells were incubated with recombinant FTP in the concentration range of 0.001–1 mg/ml DMEM.

Cell viability was determined at 72 h using the PrestoBlue Cell Viability Reagent (Invitrogen). The ability of FTP to induce 3T3 cell migration into a denuded area was evaluated using the scratch wound assay [31]. Triplicate test wells received 50 µg/ml FTP and 5 µg/ml mitomycin-C, while control wells received the vehicle alone. Three representative images from each of the scratched areas under each condition were photographed to estimate the relative migration cells. Cell images were recorded at 0, 8, 12 and 24 h post-treatment and the data further analysed with ImageJ 1.42q software (National Institutes of Health, U.S.A.) to measure the distance travelled by cells into the wound and the percentage migration at each time point. The percentage of cell migration was calculated as follows:

$$\text{Scratch closure rate}=\left[\frac{{\text{D}}_{0}-{\text{D}}_{n}}{{\text{D}}_{0}}\right]\times 100\%$$

where, *D*_0_ is the average initial distance between both sides of the scratch and *D*_n_ is the average distance between both sides of the scratch at the measured time. The experiments were performed in triplicate in two independent assays.

#### Crystallization, data collection and processing

Purified FTP was dissolved in 20 mM Tris/HCl pH 8.0 (approximately 10 mg/ml), the solution was centrifuged (12000×***g*** for 10 min) to remove any insoluble aggregates and the supernatant (200 nl drops; 100 nl FTP solution plus 100 nl well solution) screened for crystallization conditions with the JCSG-*plus*™ HT-96 Kit (Molecular Dimensions, Suffolk, U.K.) [32] using the Mosquito Crystal robot (TPP Labtech, Hertfordshire, U.K.). Crystals with high diffraction quality were obtained in condition D12 (0.04 M potassium phosphate monobasic, 16% (w/v) PEG 8000 and 20% (v/v) glycerol). Selected crystals were mounted in loops before flash-cooling in a nitrogen gas stream at 100 K. For FTP–mannose and FTP–glucose complexes, glycerol was removed from the above condition to avoid competing with the ligands, and 20 mM mannose or 100 mM glucose was added.

X-ray data collection for Apo-FTP was done at station I02, Diamond Light Source (DLS, Didcot, U.K.). Data were processed using *xia*2 [33] at DLS and the solvent content (46%) was estimated with the *MATTTHEWS_COEF* program. X-ray diffraction data for FTP–mannose and FTP–glucose complexes were collected at station I24, DLS and the data were indexed and integrated in the space group P3_1_21 by DIALS [34], while data reduction were performed using the program Aimless [35] from CCP4 suite [36].

#### Structure determination

The crystal structure of Apo-FTP was determined initially by molecular replacement using the program *Phaser MR* [37] in the *CCP4* suite with the structure of CMB lectin monomer from *Artocarpus integer* (PDB ID: 4akd) as a search model. Several rounds of manual rebuilding were then performed with *Coot* [38], followed by rounds of stereochemically restrained refinement using *REFMAC* [39].

Complex structures were determined by molecular replacement with the program *Phaser MR* using the Apo-FTP structure (PDB ID: 5krp) as search model. Model building and refinement was performed using *Coot* and *REFMAC* as above. For the FTP–glucose and FTP–mannose complexes the twin operator -*h*, -*k*, -*l* were used during refinement in *REFMAC*. The monosaccharides were introduced near the end of refinement by use of *Coot* and again underwent multiple rounds of manual rebuilding and refinement using *Coot* and *REFMAC*. Well-refined structures were submitted to *PDB_REDO* [40] for final optimization of the refinement. Omit maps were generated using the program Composite omit map in the Phenix program suite [41]. Figure 3C,D were prepared by using CueMol (Molecular Visualization Framework; http://www.cuemol.org/). Parameters for data collection, data processing and structure determination are shown in Table 1. The co-ordinates and structure factors have been deposited in the RCSB Protein Data Bank with the accession code of 5krp, 5tqz and 5m6o for the Apo-FTP, FTP–glucose and FTP–mannose complexes respectively.

#### MD simulation

Atomic co-ordinates were obtained from the crystallographic structures of FTP–mannose and FTP–glucose and submitted to the H++ program to compute the protonation state of the residues at pH = 7.4. Lysines, arginines, aspartic and glutamic acids were all ionized, while the δ and ε nitrogens of His^57^ and His^147^ received protons at both the positions for all the monomers. The atomic co-ordinates of FTP with the modifications proposed by H++ [42] were used to compose a simulation system in a dodecahedron format box with a diameter of 12.98 nm. This was filled by 47339 water molecules in the simple point charge (SPC) model [43] with four Na^+^ ions used to neutralize the system. The force field used to model FTP protein and mannose/glucose was GROMOS53A6 [44] adapted and designated asGROMOS 53A6GLYC [45]. The package GROMACS 5.0.4 was used to perform MD simulation [46–52].

#### MD parameters

The system energy minimization protocol includes a molecular mechanics step using the conjugate gradient algorithm. Then, successive MDs were performed in NVT ensemble, in which the number of atoms, volume and temperature remain constant, with frozen atomic positions of sugar and FTP protein plus use of smaller integration time-steps increasing from 0.0005, 0.001 and 0.002 ps to 10 ps to reduce bad contact and correct orientation of water molecules. The procedure was repeated with the unfrozen atomic protein and sugar positions. The final step was to retake the previous MD steps, but using the NVT ensemble [53]. The temperature in the system was controlled at 298 K by V-rescale thermostat [54] and the pressure was regulated by the Berendsen barostat at 1 atm. The SETLLE and LINCS algorithms were used to maintain the geometry of the solvent and solutes [49,55]. In addition, short-range and long-range interactions were considered within a cut-off radius (Rc =1.4 nm), while necessary corrections for the electrostatic potential were made using Particle Mesh Ewald [56] and for van der Waals using the cut-off method (Rc =1.4 nm). The trajectory was acquired during 200 ns of MD for later analysis. RMSD was obtained between the first frame of reference FTP, with other structures collected throughout the simulation. The RMSD was always preceded between the maximum superposition of Cα atoms of two structures. The RMSD of FTP was calculated from each of the four tetramer monomers (Chain A, Chain B, Chain C and Chain D) and the loop around Leu^90^ defined between residues 84 and 97 (loop) in each of the four monomers (L-Chain A, L- Chain B, L-Chain C and L-Chain D).

HBs between sugars (mannose and glucose) and FTP and water were determined as the mean number of HBs formed between pairs of sugar atoms with backbone (bb) and side chain (sc) atoms of the protein and with molecules of water. In each of the configurations obtained in the MD simulation, the HB was identified by the geometric criterion proposed by IUPAC [57] with X–H^...^Y angle is >165° and H^...^Y is <0.3 nm. For the purpose of calculating the geometry the pair, X–H is termed as a dipole and the second dipole is defined by Y–Z, where Z is the previous atom bound directly to Y. Finally, HBs between amino acid backbone (N–H) and carbonyl groups (C=O) are denoted as N–H^...^O=C [45].

## Supporting information

Supporting Video 1 Supplementary Video Files S1

Supporting Video 2 Supplementary Video Files S2

## Abbreviations

CMB: champedak mannose binding
Cα: α carbon
DLS: Diamond Light Source
FTP: frutapin
HB: hydrogen bond
IIP: intermolecular interaction potential
MCA: minimum concentration for agglutination
MD: molecular dynamics
SUMO: small ubiquitin-like modifier
ULP1: Ubl-specific protease 1
